# Detection of antibodies to citrullinated tenascin-C in patients with early synovitis is associated with the development of rheumatoid arthritis

**DOI:** 10.1136/rmdopen-2016-000318

**Published:** 2016-12-01

**Authors:** Karim Raza, Anja Schwenzer, Maria Juarez, Patrick Venables, Andrew Filer, Chris D Buckley, Kim S Midwood

**Affiliations:** 1 Institute of Inflammation and Ageing, University of Birmingham, Birmingham, UK; 2 Department of Rheumatology, Sandwell and West Birmingham Hospitals NHS Trust, Birmingham, UK; 3 Kennedy Institute of Rheumatology, Nuffield Department of Orthopaedics, Rheumatology and Musculoskeletal Sciences, University of Oxford, Oxford, UK

**Keywords:** Early Rheumatoid Arthritis, Synovitis, Ant-CCP

Early treatment of rheumatoid arthritis (RA) results in more effective disease suppression and can be key to a successful patient response. However, not all people who exhibit early synovitis develop RA; for example, in some, synovial inflammation resolves spontaneously.^1^ The factors that drive RA development remain unclear and clinical tools to predict RA development are imperfect.

Tenascin-C is a proinflammatory matrix molecule that is absent from healthy joints but highly expressed in the joints of patients with RA.^2^ ^3^ We identified an immunodominant peptide in citrullinated tenascin-C, cTNC5, antibodies against which are detected in around half of the patients with RA, and can be found years before disease onset in some individuals.^4^ Here, we sought to determine if anti-cTNC5 antibodies can discriminate among people with early synovial inflammation those who develop RA and those with other outcomes.

Sera from 263 patients in the Birmingham early arthritis cohort were analysed. Patients were disease-modifying antirheumatic drug (DMARD) naïve with clinically apparent synovitis of ≥1 joint and with inflammatory joint symptoms of ≤3 months’ duration. Patients were followed for 18 months to ensure development of full disease phenotype and to allow any resolving arthritis time to resolve. At 18 months, patients were assigned to the following outcome categories: persistent RA according to the American College of Rheumatology (ACR) 2010 criteria^5^ (RA, n=101), persistent non-RA arthritis (PNRA, n=66) and resolving arthritis (no clinically apparent joint swelling, no DMARD/steroid use in the previous 3 months, n=96). Demographic and clinical parameters were recorded, and patients with RA divided into anti-cyclic citrullinated peptide (anti-CCP) antibody positive and negative subsets.^6^ ^7^ Antibodies recognising cTNC5 or the non-citrullinated control peptide (rTNC5) were analysed by ELISA as described.^4^

Anti-cTNC5 antibodies were found in 40.6% of people with early synovitis who went on to develop RA, but were detected in a low proportion of people who developed PNRA (6.1%), or whose disease resolved (3.1%). No significant antibody response to rTNC5 was detected (p=0.527) (table 1, see online supplementary figure S1). Anti-cTNC5 antibodies were significantly more prevalent in anti-CCP antibody positive compared with anti-CCP antibody negative patients with RA (81.3% vs 3.8%, p<0.0001) (table 1). Anti-cTNC5 antibody levels were higher in patients with anti-CCP antibody-positive RA (193.1±449.8 arbitrary units (AU)) compared with patients with anti-CCP antibody-negative RA (3.56±3.30 AU), PNRA (19.42±122.7 AU) and resolving arthritis (6.60±28.02 AU) (ANOVA p<0.0001). While anti-cTNC5 was not better at predicting the development of RA than anti-CCP antibody (specificity; sensitivity: 40.6%; 95.7% (cTNC5), 47.5%; 98.8% (CCP)), anti-cTNC5 did detect a subset of people who developed RA who were not anti-CCP antibody positive (3.8%). Patients with anti-cTNC5 antibody-positive RA were more frequently anti-CCP antibody and rheumatoid factor (RF) positive than anti-cTNC5 antibody-negative patients (table 2).

**Table 1:** Demographic, clinical and laboratory characteristics of patients in each outcome group

	Anti-CCP negative RA (n=53)	Anti-CCP positive RA (n=48)	Persistent non-RA (n=66)	Resolving arthritis (n=96)	p Value
Female, n (%)	27 (50.9)	31 (64.6)	37 (56.1)	46 (47.9)	0.274
Age (years)	55.6±15.7	55.5±14.4	52.1±18.9	45.9±16.8	<0.0001
Symptom duration (days)	52.4±21.4	55.3±21.7	56.4±21.5	45.3±20.8	0.005
CRP (mg/dL)	10 (0–39)	17.5 (6–43.8)	20.5 (7.5–35.3)	7 (0–17)	<0.0001
ESR (mm/hour)	18 (11.5–44.5)	27.5 (18.3–51.3)	21.5 (7.8–45.8)	12.5 (5–27)	<0.0001
DAS28 (CRP)	4.4±1.4	4.4±1.4	3.6±1.2	2.8±1.3	<0.0001
DAS28 (ESR)	4.6±1.5	4.7±1.6	3.6±1.8	2.9±1.5	<0.001
Smoking, n (%)					0.07
Ever smoker	28/49 (57.1)	27/47 (57.4)	26/64 (40.6)	35/89 (39.3)	
Never-smoker	21/49 (42.9)	20/47 (42.6)	38/64 (59.4)	54/89 (60.7)	
Anti-CCP positive, n (%)	0 (0)	48 (100)	1 (1.5)	1 (1.0)	<0.0001
RF IgG positive, n (%)	9 (17)	44 (91.7)	5 (7.6)	10 (10.4)	<0.0001
RF IgA positive, n (%)	7 (13.2)	26 (54.2)	5 (7.6)	10 (10.4)	<0.0001
Anti-cTNC5 positive, n (%)	2 (3.8)	39 (81.3)	4 (6.1)	3 (3.1)	<0.0001
Anti-rTNC5 positive, n (%)	1 (1.9)	1 (2.1)	3 (4.5)	1 (1.0)	0.527

Data are shown as number (percentage), mean±SD, or median (IQR) as appropriate. Comparisons have been performed with χ^2^, analysis of variance (ANOVA) and Kruskal-Wallis tests for categorical, parametric continuous and non-parametric continuous data, respectively.

CCP, cyclic citrullinated peptide; CRP, C reactive protein; cTNC, citrullinated tenascin-C; DAS, disease activity score; ESR, erythrocyte sedimentation rate; RA, rheumatoid arthritis; RF, rheumatoid factor.

**Table 2:** Characteristics of patients with RA with and without anti-cTNC5 antibodies

	Anti-cTNC5 negative RA (n=60)	Anti-cTNC5 positive RA (n=41)	p Value
Female, n (%)	33 (55)	25 (60.1)	0.682
Age (years)	55.2±16.1	56.1±13.3	0.785
Symptom duration (days)	52.3±21.5	56±21.5	0.400
CRP (mg/dL)	10.5 (0–43)	18 (6–39)	0.062
ESR (mm/hour)	18 (11–45)	25 (19–46)	0.372
DAS28 (CRP)	4.26±1.4	4.55±1.4	0.320
DAS28 (ESR)	4.51±1.5	4.82±1.6	0.320
28 TJC	7.22±6.5	9.1±10.4	0.267
28 SJC	7.6±7.2	6.9±5.5	0.595
Smoking, n (%)			
Ever smoker	34/56 (60.7)	21/40 (52.5)	0.682
Never-smoker	22/56 (39.3)	19/40 (47.5)	0.374
Anti-CCP positive, n (%)	9 (15)	39 (95.1)	<0.0001
RF IgG positive, n (%)	16 (26.7)	37 (90.2)	<0.0001
RF IgA positive, n (%)	10 (16.6)	23 (56.1)	<0.0001

Data are shown as number (percentage), mean±SD, or median (IQR) as appropriate. Comparisons have been performed with χ^2^, Student's t-test and Mann Whitney U test for categorical, parametric continuous and non-parametric continuous data, respectively.

CCP, cyclic citrullinated peptide; CRP, C reactive protein; cTNC, citrullinated tenascin-C; DAS, disease activity score; ESR, erythrocyte sedimentation rate; RA, rheumatoid arthritis; RF, rheumatoid factor; SJC, swollen joint count; TJC, tender joint count.

Together these data reveal that detection of anti-cTNC5 antibodies in the sera of people with early synovitis is associated with the development of RA. While similar numbers of people who developed RA were positive for anti-cTNC5 antibodies, as were positive for anti-CCP antibodies, these two groups did not entirely overlap; we identified a subset of anti-CCP antibody negative, anti-cTNC5 antibody positive patients (3.8%). This study therefore does not support replacing CCP analysis with cTNC5 analysis to accurately predict which patients presenting with early joint inflammation will go on to develop RA. However, a combined analysis of CCP, cTNC5 and other citrullinated antigens may increase the number of people who can be diagnosed with RA at this early stage. Although a small proportion of the total patient number, when translated into the number of people who might otherwise be missed, this could bring significant clinical benefit.

Analysis of distinct subsets of antibodies recognising different citrullinated peptides (anti-citrullinated peptide antibodies, ACPA) can yield information that is not possible to derive using artificial CCP peptides to detect ACPA. Arising before overt clinical symptoms, ACPA have the potential to reveal insights into disease aetiology. For example, gene/environment (major histocompatibility complex shared epitope and smoking) interactions are strongest in people who are dual positive for antibodies against citrullinated α-enolase and for antibodies recognising citrullinated vimentin.^8^ We previously found that anti-cTNC5 antibody positivity did associate with smoking in the EIRA (Epidemiological Investigation of Rheumatoid Arthritis) cohort; however, this link was weaker than that observed for APCA recognising citrullinated enolase.^4^ Here, we observed that the ratio of ever smoker versus never-smoker, while only slightly decreased in cTNC5-positive patients (52.5%:47.5%), was substantially decreased in anti-cTNC5 antibody-negative patients (60.7%:39.3%), although no significant association between anti-cTNC5 antibody status and smoking was observed. These data suggest that further studies investigating whether anti-cTNC5 antibody positivity could mark a serologically distinct subset of people who will develop RA would be of interest.

Finally, emerging evidence indicates that ACPA actively contribute to inflammation, and can directly drive tissue destruction that is the hallmark of established RA. Uncovering the identity of peptides that give rise to ACPA has started to reveal more about these mechanisms underlying disease pathogenesis. For example, immune complexes containing anticitrullinated fibrinogen antibodies signal to induce proinflammatory cytokine synthesis, and antibodies to citrullinated vimentin provoke osteoclastogenesis and bone erosion.^9^ ^10^ However, little is known about the contribution of the autoantibody response to the events that drive early synovitis onto RA. Our finding that anti-cTNC5 antibodies were raised only in people whose synovitis progressed to RA opens the door for further work investigating whether these antibodies play a causal role in driving the differentiation of early joint inflammation towards persistent RA and away from disease resolution.
